# Undifferentiated Carcinoma of the Pancreas With Osteoclast-Like Giant Cells Localized in the Main Pancreatic Duct Without Extraductal Invasion: A Case Report and Literature Review

**DOI:** 10.7759/cureus.80592

**Published:** 2025-03-14

**Authors:** Yosuke Takahashi, Masayoshi Hioki, Kyotaro Ohno, Hiroshi Sadamori, Norihisa Takakura

**Affiliations:** 1 Department of Gastroenterological Surgery, Okayama University Graduate School of Medicine, Dentistry, and Pharmaceutical Sciences, Okayama, JPN; 2 Department of Surgery, Fukuyama City Hospital, Fukuyama, JPN; 3 Department of Pathology, Fukuyama City Hospital, Fukuyama, JPN

**Keywords:** curative resection, intraductal growth, long-term survival, main pancreatic duct, undifferentiated carcinoma with osteoclast-like giant cells

## Abstract

Undifferentiated carcinoma with osteoclast-like giant cells (UC-OGC) is a rare pancreatic tumor and typically presents as a giant hypervascular tumor with rapid growth and intratumoral hemorrhage. UC-OGC localized in the main pancreatic duct (MPD) without extraductal invasion is extremely rare and difficult to diagnose preoperatively. Although the tumor rapidly increases in size and often becomes too large for resection, patients with UC-OGC who undergo curative resection show a better prognosis than those with other types of undifferentiated carcinomas and pancreatic ductal adenocarcinoma (PDAC). We must recognize that UC-OGC could present as an MPD-localized tumor and, therefore, should not miss the chance for resection. In this study, we present an unusual case of UC-OGC that was completely localized in the MPD without extraductal invasion.

A 77-year-old Japanese man presented to our hospital with excessive thirst. Blood tests showed elevated glycosylated hemoglobin levels (11.9%). Carcinoembryonic antigen, carbohydrate antigen 19-9, Duke pancreatic monoclonal antigen type 2, Span-1, and neuron-specific enolase levels were within normal ranges. Contrast-enhanced computed tomography (CT) showed a 22-mm indistinct nodule with prolonged enhancement in the pancreatic body and dilatation of the distal MPD. Fluorine-18-fluorodeoxyglucose positron-emission tomography with CT showed uptake at the nodule but no evidence of metastasis. Endoscopic ultrasonography showed that the tumor was a heterogeneous hypoechoic nodule localized in the MPD. Pancreatic juice cytology indicated atypical cells but no evidence of malignancy. We suspected PDAC, acinar cell carcinoma, or an intraductal tubulopapillary neoplasm. We performed laparoscopic distal pancreatectomy and splenectomy, along with lymph node dissection. Histopathological examination revealed a 30-mm intraductal tumor with intratumoral hemorrhage, fibrosis, and angiogenesis. The tumor was composed of atypical spindle cells that were partly positive for cytokeratin AE1/3, CAM5.2, and vimentin and scattered osteoclast-like multinucleated giant cells that were positive for CD68. The tumor was completely localized to the MPD without extraductal invasion or lymph node metastasis. The patient received tegafur, gimeracil, and oteracil potassium as postoperative adjuvant chemotherapy for six months and has been recurrence-free for more than five years. UC-OGC demonstrates rapid growth; however, a good prognosis can be expected with curative resection.

## Introduction

Undifferentiated carcinoma with osteoclast-like giant cells (UC-OGC) is a rare type of pancreatic tumor with unique clinical and pathological features. UC-OGC accounts for 0.8% of all pancreatic tumors and 1.4% of pancreatic ductal carcinomas [1]. UC-OGC was previously classified as a subtype of UC; however, the latest classification published by the World Health Organization in 2019 defined it as being completely distinct from UCs owing to differences in biological behavior [2,3]. Although UC-OGC is often reported to show a rapid expansive growth pattern with intertumoral hemorrhage and necrosis similar to UC and is found as a hypervascular giant tumor, UC-OGC has a better curative resection rate and prognosis than UC [1,4-6].

In this study, we describe a morphologically unusual case of UC-OGC that was completely localized in the main pancreatic duct (MPD) without extraductal invasion. No evidence of recurrence has been observed for more than five years following surgery.

## Case presentation

**Figure 1:**
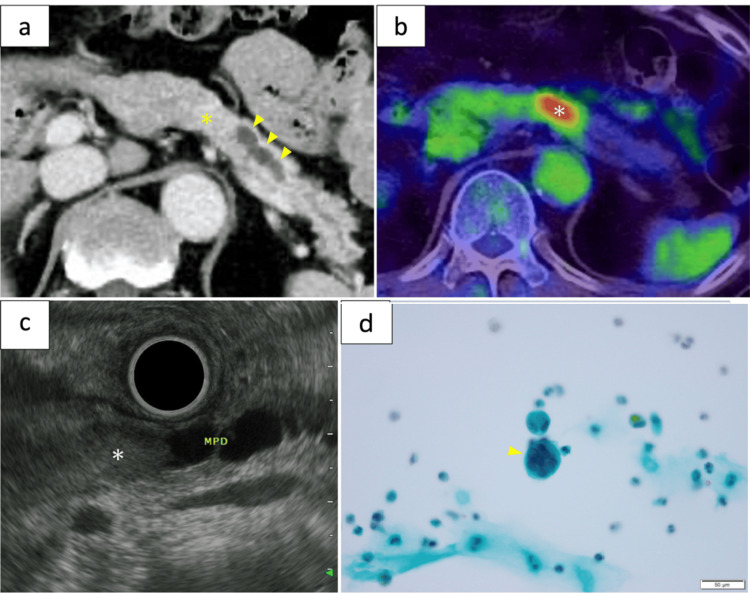
Image findings. (a) Abdominal CT (equilibrium phase) showing a 22-mm indistinct nodule (asterisk) with prolonged enhancement and a dilated distal MPD (arrowhead). (b) Fluorine-18-fluorodeoxyglucose positron-emission tomography with CT showing uptake in the nodule (asterisk). (c) Endoscopic ultrasonography showing the tumor as a 26 × 10-mm heterogeneous hypoechoic nodule localized in the MPD (asterisk). (d) Pancreatic juice cytology showing atypical cells in four of seven samples (Papanicolaou staining ×40; arrowhead) CT: computed tomography; MPD: main pancreatic duct

A 77-year-old Japanese man presented with excessive thirst and was referred to our hospital with an elevated glycosylated hemoglobin level of 11.9% and a dilated MPD on computed tomography (CT). The patient had dermatomyositis and primary biliary cirrhosis and received medications, including steroids. Blood tests showed that the serum levels of amylase, bilirubin, carcinoembryonic antigen, carbohydrate antigen 19-9, Duke pancreatic monoclonal antigen type 2, Span-1, and neuron-specific enolase were within normal limits. Contrast-enhanced CT showed a 22-mm indistinct nodule with prolonged enhancement in the pancreatic body and dilatation of the distal MPD (Figure 1a). Fluorine-18-fluorodeoxyglucose positron-emission tomography with CT showed uptake at the nodule but no evidence of metastasis (Figure 1b). Endoscopic ultrasonography showed that the tumor was a 26 × 10-mm heterogeneous hypoechoic nodule localized in the MPD (Figure 1c). Pancreatic juice was collected via an indwelling endoscopic nasal pancreatic drainage tube for three days, and its cytology showed atypical cells in four of seven samples (Figure 1d).

**Figure 2:**
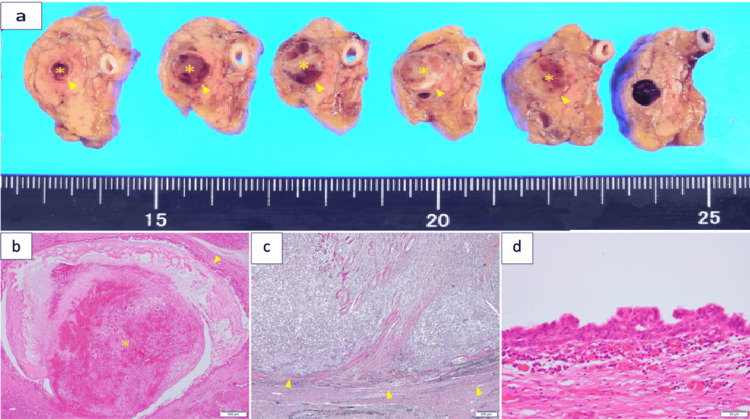
Macroscopic and histopathological findings. (a) A 30-mm whitish polypoid tumor (asterisk) located within the MPD (arrowhead). (b) A pedunculated polypoid tumor (asterisk) is completely localized in the MPD (arrowhead; H&E staining, ×2). (c) Elastica van Gieson staining of the tumor showing no extraductal invasion (arrowhead; ×4). (d) Epithelium of the dilated MPD partly exhibits cellular atypia ranging from PanIN2 to 3 (H&E staining, ×40) MPD: main pancreatic duct; H&E: hematoxylin and eosin

We suspected pancreatic ductal adenocarcinoma (PDAC), acinar cell carcinoma, or an intraductal tubulopapillary neoplasm (ITPN). We performed laparoscopic distal pancreatectomy and splenectomy, along with lymph node dissection. The pancreas from the body to the tail and the spleen were resected en bloc and fixed in 10% buffered formalin. Macroscopically, a whitish polypoid tumor measuring 30 mm was located within the lumen of the MPD (Figure 2a).

**Figure 3:**
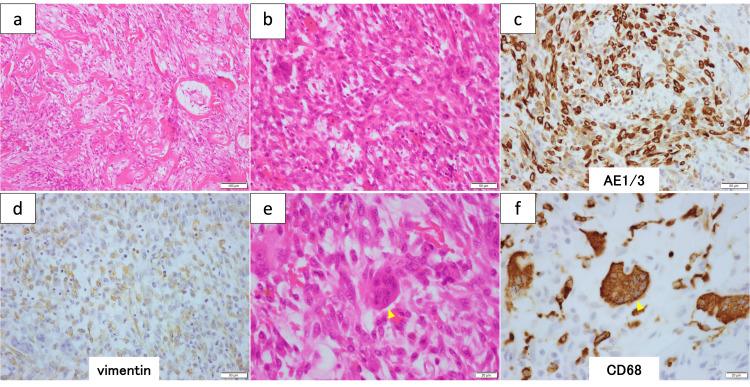
Immunohistopathological findings indicating UC-OGC. (a) Tumor showing intratumoral hemorrhage, fibrosis, and angiogenesis (H&E, ×10). (b-d) Tumor is primarily composed of atypical spindle cells that are partly positive for the epithelial markers cytokeratin AE1/3 or CAM5.2 or for the mesenchymal marker vimentin (H&E and immunohistochemistry, ×20). (e,f), The tumor contains scattered osteoclast-like multinucleated giant cells (arrowhead) positive for CD68 (H&E and immunohistochemistry, ×40) H&E: hematoxylin and eosin; UC-OGC: undifferentiated carcinoma with osteoclast-like giant cell

Histopathological examination with hematoxylin and eosin staining revealed that the tumor was composed of proliferating atypical spindle cells (SCs) and scattered osteoclast-like multinucleated giant cells with intratumoral hemorrhage, fibrosis, and angiogenesis (Figures 3a-3c). The SCs were partly positive for both cytokeratin AE1/3 or CAM5.2 and vimentin, and the OGCs were positive for CD68 on immunohistochemical examination (Figures 3d-3f).

These findings led to a diagnosis of UC-OGC. The pedunculated polypoid tumor was completely localized in the MPD and lacked extraductal invasion (Figures 2b, 2c). There was no evidence of neural or vascular invasion or regional lymph node metastasis. In the specimens, the epithelium of the dilated MPD partly exhibited cellular atypia ranging from PanIN2 to 3 (Figure 2d). Although the patient required anticoagulant therapy for portal vein thrombosis, he was discharged 43 days postoperatively. The patient received one course of adjuvant chemotherapy with tegafur, gimeracil, and oteracil potassium 100 mg/body for four weeks as adjuvant chemotherapy. This course was not repeated, owing to the development of a skin rash. No evidence of recurrence has been observed for more than five years following surgery.

## Discussion

UC-OGC was first described by Sommer and Meissner in 1954. Rosai proposed UC-OGC as a variant of a pancreatic tumor with giant cell carcinoma of the osteoclastoid in 1968 [7,8]. It is characterized by three cellular components: nonneoplastic osteoclast-like multinucleated giant cells, neoplastic mononuclear cells, and mononuclear histiocytes.

A search of PubMed (terms used: [“undifferentiated” {All Fields} AND “carcinoma” {All Fields}] OR “undifferentiated carcinoma” {All Fields} OR [“anaplastic” {All Fields} AND “carcinoma” {All Fields}] OR [“anaplastic carcinoma” {All Fields} AND “pancrea” {All Fields} OR “pancreas” {MeSH Terms}] OR “pancreas” {All Fields}) was performed, and 467 articles published between 1953 and 2024 were identified. As UC-OGC is often confused with other types of pancreatic tumors, we could not distinguish some cases as UC or UC-OGC. After excluding articles that did not include UC-OGC cases or were not written in English, more than 100 papers on UC-OGC were identified. However, only five cases of UC-OGC predominantly located within the MPD have been reported [9-13]. For these five cases and the present case, we evaluated sex, age, symptoms, tumor size, extraductal invasion, lymph node or distant metastasis, recurrence-free survival, preoperative diagnosis, and biopsy method (Table 1).

**Table 1:** The patients with UC-OGC predominantly located within the MPD UC-OGCs: undifferentiated carcinoma with osteoclast-like giant cells; NET: neuroendocrine tumor; IPMC: intraductal papillary mucinous carcinoma; IPMN: intraductal papillary mucinous neoplasm; UC: undifferentiated carcinoma; PDAC: pancreatic ductal adenocarcinoma; ITPN: intraductal tubulopapillary neoplasm; FNA: fine-needle aspiration

Author	Year	Sex	Age (year)	Symptoms	Extraductal invasion	Tumor size (cm)	Lymph node or distant metastasis	Recurrence-free survival months	Preoperative diagnosis	Biopsy method
Tezuka et al. [12]	2006	Female	68	Abdominal pain	No	4.2	No	22	IPMN	FNA
Naito et al. [9]	2009	Female	76	Abdominal pain	Unknown	1.8	No	19	UC-OGC	Pancreatic duct brush cytology
Ishii et al. [13]	2012	Male	61	No	No	3.2	Unknown	14	UC	Pancreatic juice cytology
Sato et al. [10]	2019	Female	61	No	Present	4	No	35	NET	No
Yamamoto and Sakai [11]	2021	Male	71	No	No	3.5	No	20	IPMC	No
Our case	-	Male	77	Thirst	No	2.6	No	57	PDAC, acinar cell carcinoma, or ITPN	Pancreatic juice cytology

The tumors in all six cases showed intraductal growth within the dilated MPD, and the average tumor size was 3.3 (1.8-4.2) cm. UC-OGC is often reported as a giant tumor. Notably, 80% and 50% of UC-OGCs are reportedly larger than 5 and 10 cm, respectively [14,15]. However, in all six cases, the tumors located in the MPD were relatively small, and those in four cases, including ours, were completely localized within the MPD without extraductal invasion. Three patients were symptomatic, possibly because of the predominant development of the tumor within the MPD. Other patients were asymptomatic and diagnosed during routine health checkups.

All six patients underwent surgical resection and had no lymph node or distant metastasis. The average postoperative observation period was 28 (14-57) months. As none of the patients had recurrence at the time of publication, the survival periods of UC-OGC located in the MPD would be significantly longer. Christopher et al. analyzed 423,482 patients with pancreatic cancer enrolled in the National Cancer Database and reported that the median survival period of UC-OGC was 24.8 months (n = 119), which was significantly longer than that of UC without OGCs (3.8 months; n = 739) [6]. Another study showed that UC-OGC had a better five-year survival rate and median survival period than PDAC (59.1% and 7.67 years vs. 15.7% and 1.59 years, respectively) [1]. Shiihara et al. evaluated previous articles and reported that the median overall survival period of patients with UC-OGC who underwent surgical resection (n = 113) was 48 months, which was longer than that of patients with UC. They also reported that pleomorphic cell carcinoma, a subtype of UC, showed significantly higher rates of lymph node metastasis than UC-OGC [5]. By contrast, another study reported that UC-OGC rarely showed perineural invasion or lymph node metastasis [1]. However, patients with unresectable UC-OGC and locally advanced tumors or distant metastases show an extremely poor median survival of less than six months [4,16]. Fujimoto et al. reported a case in which a 20-mm tumor at initial presentation increased to 40 mm in only 40 days and became unresectable due to the invasion of the surrounding blood vessels. The tumor rapidly increased in size and measured 18 cm after 11 months [17]. Because UC-OGC shows rapid growth, we must be careful not to miss the chance for resection to prevent a patient with curable UC-OGC from having a very poor prognosis.

Among the six patients, only one was preoperatively diagnosed with UC-OGC via pancreatic duct brush cytology. Three patients, including ours, could not be diagnosed before surgery despite performing pancreatic fluid cytology or fine-needle aspiration (FNA). For the pancreatic tumors located within the MPD, intraductal papillary mucinous neoplasm, ITPN, acinar cell carcinoma, and nonfunctional endocrine tumor are mentioned as differential diagnoses [10,12,18,19]. UC-OGC occasionally demonstrates infiltration into the MPD and intraductal growth [1]; however, tumors localized within the MPD without extraductal invasion are extremely rare. Sato et al. reported a case of UC-OGC predominantly localized within MPD, showing hyperintensity on the R2* map and in-phase signal reduction of MRI, suggesting its potential diagnostic value [10]. In a study of 15 patients with UC-OGC who underwent preoperative FNA, only six were accurately diagnosed [15]. UC-OGC is difficult to diagnose with a biopsy, even if it is located within the MPD. Recently, a new technology combining fine-needle biopsy and next-generation sequencing was reported, which is expected to improve preoperative diagnosis [20]. However, preoperative FNA increases the incidence of postoperative complications and requires careful selection [4].

## Conclusions

Although UC-OGC demonstrates rapid growth, patients can achieve a favorable prognosis with curative resection. In particular, cases confined to the MPD, as in the present case, could be associated with a favorable postoperative outcome. However, such tumors are extremely rare and challenging to diagnose preoperatively. This case highlights the importance of considering UC-OGC in the differential diagnosis of tumors predominantly localized within the MPD, particularly when biopsy results are inconclusive.

## References

[1] Muraki T, Reid MD, Basturk O (2016). Undifferentiated carcinoma with osteoclastic giant cells of the pancreas: clinicopathologic analysis of 38 cases highlights a more protracted clinical course than currently appreciated. Am J Surg Pathol.

[2] Fukushima N, Hruban RH, Kato Y (2010). Ductal adenocarcinoma variants and mixed neoplasms of the pancreas. WHO Classification of Tumours of the Digestive System.

[3] Hruban RH, Maitra A, Adsay NV (2019). Pancreatic ductal adenocarcinoma. WHO Classification of Tumours Digestive System Tumours.

[4] Wu H (2024). Undifferentiated carcinoma with osteoclast-like giant cells of the pancreas: a narrative review. Front Oncol.

[5] Shiihara M, Higuchi R, Izumo W, Furukawa T, Yamamoto M (2020). A comparison of the pathological types of undifferentiated carcinoma of the pancreas. Pancreas.

[6] Christopher W, Nassoiy S, Marcus R (2022). Prognostic indicators for undifferentiated carcinoma with/without osteoclast-like giant cells of the pancreas. HPB (Oxford).

[7] Sommers SC, Meissner WA (1954). Unusual carcinomas of the pancreas. AMA Arch Pathol.

[8] Rosai J (1968). Carcinoma of pancreas simulating giant cell tumor of bone. Electron-microscopic evidence of its acinar cell origin. Cancer.

[9] Naito Y, Kinoshita H, Okabe Y (2009). Pathomorphologic study of undifferentiated carcinoma in seven cases: relationship between tumor and pancreatic duct epithelium. J Hepatobiliary Pancreat Surg.

[10] Sato K, Urakawa H, Sakamoto K, Ito E, Hamada Y, Yoshimitsu K (2019). Undifferentiated carcinoma of the pancreas with osteoclast-like giant cells showing intraductal growth and intratumoral hemorrhage: MRI features. Radiol Case Rep.

[11] Yamamoto S, Sakai Y (2021). A case of undifferentiated carcinoma with osteoclast-like giant cells of the pancreas derived from an intraductal papillary mucinous neoplasm. Clin J Gastroenterol.

[12] Tezuka K, Yamakawa M, Jingu A, Ikeda Y, Kimura W (2006). An unusual case of undifferentiated carcinoma in situ with osteoclast-like giant cells of the pancreas. Pancreas.

[13] Ishii S, Kobayashi G, Fujita N (2012). Undifferentiated carcinoma of the pancreas involving intraductal pedunculated polypoid growth. Intern Med.

[14] Gao HQ, Yang YM, Zhuang Y, Liu P (2015). Locally advanced undifferentiated carcinoma with osteoclast-like giant cells of the pancreas. World J Gastroenterol.

[15] Reid MD, Muraki T, HooKim K (2017). Cytologic features and clinical implications of undifferentiated carcinoma with osteoclastic giant cells of the pancreas: an analysis of 15 cases. Cancer Cytopathol.

[16] Imaoka H, Ikeda M, Maehara K (2021). Risk stratification and prognostic factors in patients with unresectable undifferentiated carcinoma of the pancreas. Pancreatology.

[17] Fujimoto T, Inatomi O, Mizuno R (2018). Anaplastic pancreatic cancer diagnosed with endoscopic ultrasound guided fine needle aspiration showing hypervascular tumor: a case report. Medicine (Baltimore).

[18] Akatsu T, Wakabayashi G, Aiura K (2004). Intraductal growth of a nonfunctioning endocrine tumor of the pancreas. J Gastroenterol.

[19] Hashimoto M, Matsuda M, Watanabe G, Mori M, Motoi N, Nagai K, Ishibashi M (2003). Acinar cell carcinoma of the pancreas with intraductal growth: report of a case. Pancreas.

[20] Imaoka H, Sasaki M, Hashimoto Y, Watanabe K, Ikeda M (2019). New era of endoscopic ultrasound-guided tissue acquisition: next-generation sequencing by endoscopic ultrasound-guided sampling for pancreatic cancer. J Clin Med.

